# Tooth size discrepancy in a Libyan population, a cross-sectional study in schoolchildren

**DOI:** 10.4317/jced.51819

**Published:** 2015-02-01

**Authors:** Iman Bugaighis, Divakar Karanth, Ali Borzabadi-Farahani

**Affiliations:** 1 BDS, MDS, PhD. Department of Orthodontics, Dental Faculty, Benghazi University, Benghazi, Libya; 2 BDS, MDS, M Orth RCS. The Multispecialty Dental Clinic, Porvorim, Bardez, Goa, India; 3 MScD, M Orth RCS, Fellowship Craniofac Orth (CHLA/USC). Orthodontics, Warwick Dentistry, Warwick Medical School, University of Warwick, Coventry; Maxillofacial Unit and Orthodontics, Northampton General Hospital, Northampton, UK

## Abstract

Objetives: The aim of this cross-sectional study was to investigate the tooth size discrepancy (TSD) in a group of Libyan schoolchildren, and to compare TSD between sexes. Material and Methods: The sample comprised 333 Libyan schoolchildren (162 males with a mean (SD) age of 14.4 (1.1) years, and 171 females with a mean age of 14.1 (1.1) years). Anterior and overall TSD ratios were computed using descriptive statistics. Sex differences were statistically assessed using an independent t-test (P<0.05). Results: Males showed significantly wider MD tooth width compared to females (P<0.05), except for the maxillary first premolars and mandibular central incisors. There were significant differences (P<0.05) between the paired (right and left sides) tooth measurements except for the maxillary and mandibular central and lateral incisors as well as mandibular canines. The mean (SD) for overall and anterior TSD ratios were 91.3% (2.1) and 78.2% (2.6), respectively, with no significant sex differences for both variables (P> 0.05). The percentages of participants showing more than 2 SD variation for the anterior and overall ratios comprised 3% and 4.2% of the total sample, respectively. Conclusions: The anterior and overall TSD ratios for the examined subjects were established and showed no significant sexual dimorphism.

** Key words:**Tooth size discrepancy, Libyan, schoolchildren.

## Introduction

An ideal interarch relationship with normal overjet and overbite requires the existence of a desirable ratio of the maxillary versus mandibular mesio-distal [MD] crown width (1). This conclusion was based on Bolton’s investigation (1) of the intermaxillary correlation of 55 subjects with normal occlusion. Bolton observed that in his subjects, the overall ratio obtained by dividing the sum of the MD width of all the mandibular permanent teeth except the second and third molars by the sum of the MD width of the corresponding 12 maxillary teeth was 91.3±0.26 [standard deviation [SD]] per cent, and the anterior ratio obtained by dividing the sum of the mandibular six anterior teeth by the corresponding maxillary teeth was 77.2±0.22 [SD] per cent. Subsequently, many studies reported variation of those ratios among different interarch occlusal categories, and various ethnicities (2-8). However, Othman and Harradine (9) concluded that clinically significant variations were not expected to be observed among different races. Most of the conducted research was undertaken in subjects attending orthodontic clinics (4-7,10). Several studies examined subjects from the general public (3,11,12) with variation in the number of the examined groups ranging between 55-710.

There is a lack of consensus in the literature concerning sex differences in relation to tooth size discrepancy [TSD]. While many studies reported a significant difference in TSD between males and females (6,8). Others did not observe sexual dimorphism in their examined populations (4,5,7,10,13,14). Othman and Harradine (9) concluded in their literature review that there were no significant differences in Bolton’s ratios between males and females, although, a few of the reviewed studies reported larger male tooth size compared to females. The prevalence of significant TSD in the general population has been quoted to be around 5%. Bolton (15) reported that there was a wide range of MD tooth width by which a normal occlusion could be attained. A clinically relevant TSD is defined as a value of more or less than normal by 2 SD (4,15).

The literature search on key words such as tooth size discrepancy, Bolton’s ratio, Libyans, and MD tooth width revealed that there were no published studies on TSD for Libyan subjects [July 2014]. Therefore, the present study was designed to determine the mean MD tooth width and Bolton’s anterior and overall ratios, as well as to explore the possible existence of any sexual dimorphism in a representative sample of the Libyan schoolchildren living in the Benghazi city.

## Subject and Methods

Ethical approval was secured from the Ministry of Health in Benghazi-Libya and parents of students were informed. The sample was randomly selected from children attending intermediate schools in Benghazi city. Benghazi is the second largest city in Libya with approximately 1,000,000 residents. The total number of students attending these schools was 43,881 [22,248 females and 21,633 males]. Four intermediate schools obtained from the directory of the Ministry of Education in the city were chosen randomly from each of the five geographic districts [Central, Northern, Southern, Western and Eastern]. A stratified sampling approach was followed where the number of subjects recruited from each district varies along with the total number of students to ensure fair representation of the targeted population.

A record of the children’s names in each classroom was obtained; each fifth child was examined to assure randomization. The students who fulfilled the inclusion criteria were included in the study. Nine hundred students [453 males and 447 females] aged 12-17 years; attending intermediate schools were examined at the school premises by one examiner [I.B]. The participants were of Libyan descent for at least two generations, with no craniofacial abnormalities and none had undergone previous orthodontic treatment. All permanent teeth were fully erupted up to the first molar, with no caries or restorations, and no micro or macrodontia that might interfere with accurate assessment; Only 343 Libyan schoolchildren fulfilled the reported requirements. A full detail of the current group is reported elsewhere.

Upper and lower arch Alginate impressions [ALGINKID, Italy] and wax bite registrations were recorded and then casted on the same morning with dental stone. All models were checked and numbered. All the measurements were extracted using an electronic digital caliper of an accuracy of 0.01mm [0-150 mm Digital Calliper/Lin 48772] by one operator [D.K]. An Excel spreadsheet file was prepared including all the recorded tooth measurements.

## Method Error

Thirty randomly selected dental casts were reexamined at two-week interval to assess intraoperator and interoperator occlusal trait’s measurement reproducibility. The paired t-test revealed no significant differences between both measurements at *P*>0.05. Intra-class Correlation Coefficient [ICC] was found to be greater than 0.90 indicating an excellent level of reproducibility between both trials.

## Statistical Analysis

The data were analyzed using the SPSS version 17 [Chicago, IL, USA]. The Shapiro–Wilk test was used to investigate the distribution of the data and Levene’s test to explore the homogeneity of the variables. Ten cases were displayed as outliers and were excluded from the analysis leaving the total number at 333 subjects [162 males and 171 females with a mean age of 14.4 [1.1] years and 14.1 [1.1] years, respectively]. Subsequently, Shapiro-Wilk test revealed that the data was normally distributed. The paired t - test was undertaken to detect significant differences between paired tooth measurements [right and left side]. The unpaired student t-test was applied to explore significant sex differences. The level of significance was set at *p*<0.05.

## Results

The sample sex distribution is displayed in  Table 1. A normal occlusion was registered in 4.2%. The frequency of malocclusions was 95.8%. Class I, Class II division 1, Class II division 2, and Class III malocclusions were registered in 66.7%, 21.6%, 3.6%, and 3.9%, respectively.

**Table 1:**
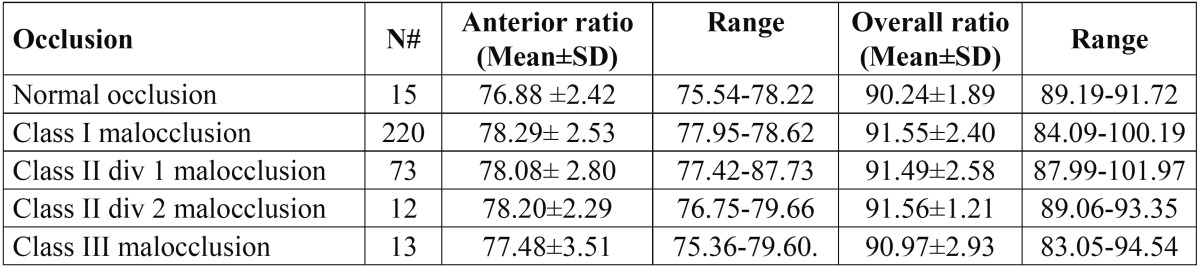
Mean, standard deviation (SD), and the range of ratios in each malocclusion group.

- Sexual dimorphism in MD tooth measurement and differences in paired tooth measurements:

The mean [SD] and statistical comparisons of the MD tooth width for males and females are shown in table 2. Males showed significantly wider MD tooth width compared to females [*P*<0.05], except for the Maxillary first premolars and mandibular central incisor. There were significant differences between the paired tooth measurements [*P*<0.05], except for the maxillary and mandibular central and lateral incisors and mandibular canines [*P*>0.05] ( Table 2).

**Table 2:**
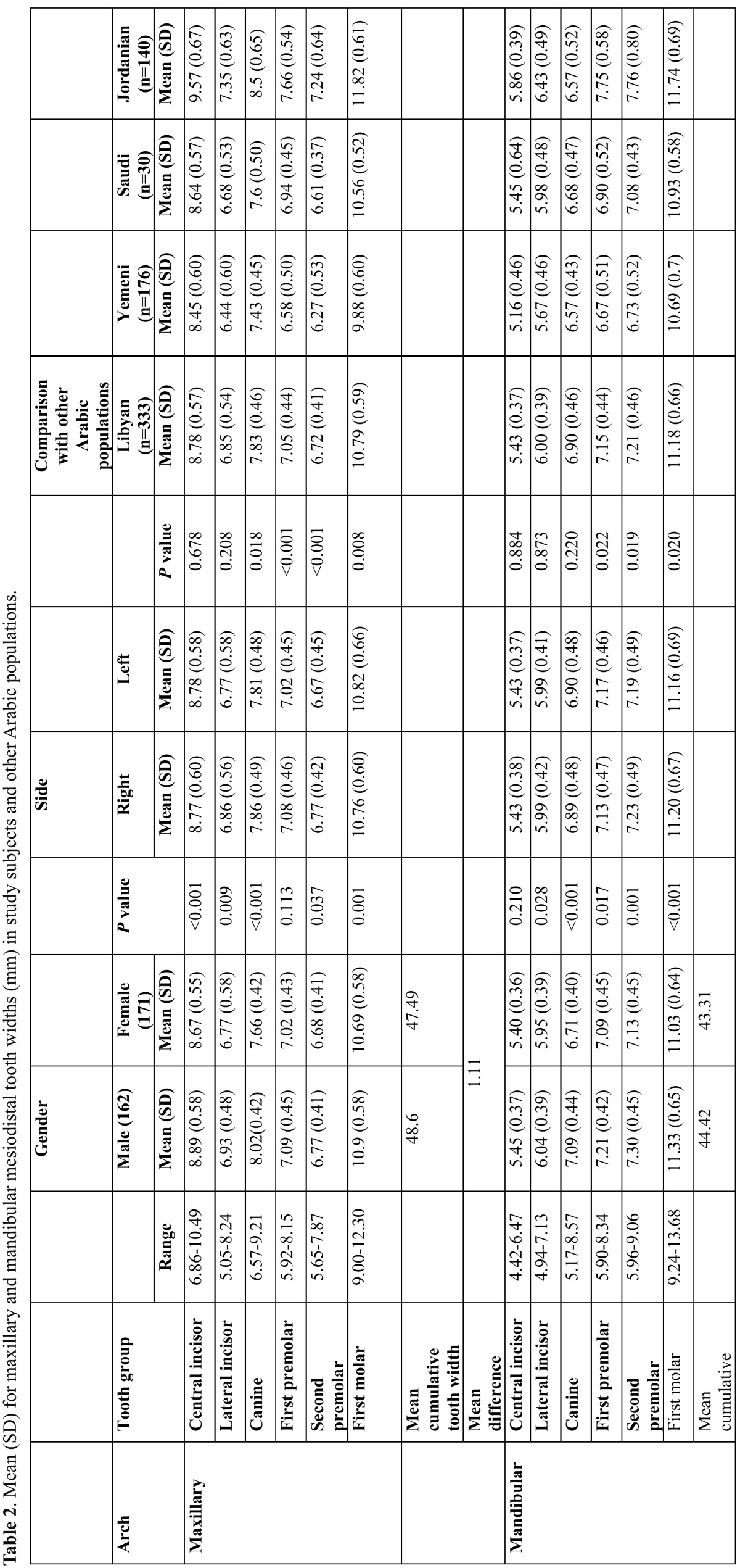
Mean (SD) for maxillary and mandibular mesiodistal tooth widths (mm) in study subjects and other Arabic populations.

- Comparison of TSD in Libyans and other populations:

 Table 3 compares the anterior and overall tooth size ratios for Libyans with other populations. The anterior and overall ratios in Libyans were 78.2±2.6% and 91.3±2.1%, respectively.

**Table 3:**
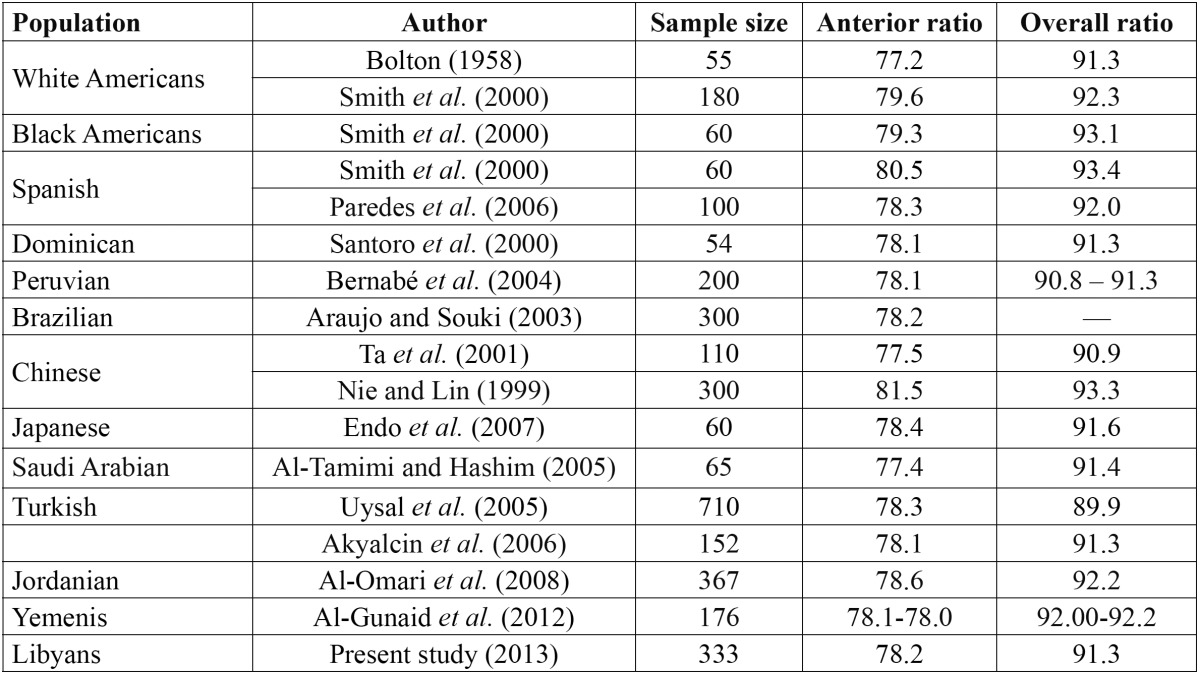
Anterior and overall tooth size discrepancy (%) in different populations.

- TSD and sex:

 Table 4 shows that there were no significant sex differences [*P* >0.05] between the anterior TSD ratio in males [78.0±2.4%] and females [78.3± 2.8%]. Further, there was no significant difference [*P* > 0.05] between the overall TSD ratios in males [91.4±2.2%] and females [91.2 ±2.1%]. The TSD frequency of 1 SD, 2 SD, and more than 2 SD from Bolton’s mean for anterior and overall ratios are shown in  Table 4. The frequency of anterior and overall mean ratios with significant deviation [> 2 SD] comprised 3% and 4.2% of the total sample, respectively.

**Table 4:**
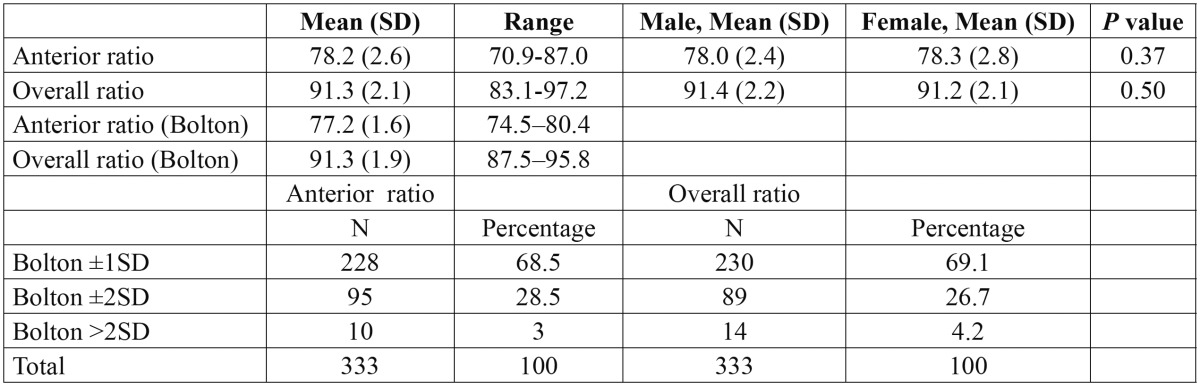
Total, anterior and overall tooth size discrepancy in the sample, in males (162) and females (171) (%), and the corresponding *P* values. The second part shows the distribution of subjects with anterior and overall tooth size discrepancies (%).

## Discussion

The finishing stage of orthodontic treatment requires detailed and refined tooth positioning, which is difficult to be reached in the presence of a tooth size discrepancy [TSD]. This research was undertaken on a representative sample of Libyan subjects attending schools in Benghazi city. A relatively younger age group was selected to minimize the influence of tooth wear on the study outcome. Significant differences were noted between the MD tooth widths except for the MD tooth width of the upper and lower central and lateral incisors, as well as the lower canines. However, these differences were not clinically significant and considered within the range of measurement errors. Definite differences in paired MD tooth width were reported in a few studies (16,17). On the other hand, similar paired MD tooth measurements were observed in Yemeni’s population (2) and in Saudi subjects (18) from Arabic societies, in addition to subjects from other ethnicities (19).

Different conclusions can be taken from studies of the relationship between race/sex and tooth size. Lavelle *et al.* (8) reported that the MD crown width in Nigroids was greater than in Caucasians. Bishara *et al.* (20) suggested that Egyptian male’s canines and first molars, as well as the sum of the upper canines and first and second premolars were larger in Egyptian females. Gunaid *et al.* (2) noticed that Yemeni’s males had significantly larger teeth than females, except for the sizes of the upper lateral incisors. Al-Omari *et al.* (3) found larger tooth sizes for most Jordanian male teeth compared to females. This study demonstrated that Libyan male teeth were significantly wider in females, except for the upper first premolars and lower central incisors. This difference was also observed in the cumulative MD tooth size widths, where male values exceeded those of the females by 1.11 mm in each of the upper and lower teeth. The present finding was in line with those observed in other Arabian groups of Iraqis (21), Jordanians (3,16), and Saudis (18), as well as for subjects from other races (22,23). The present study showed that the examined Libyan subjects had wider teeth compared to the published figures of other Arabian populations [Yemeni’s (2) and Saudi’s (24)], but had narrower teeth compared to Jordanians (16), except for the lower canines, which were wider in the Libyan group ( Table 2). These differences might be due to racial differences.

The anterior TSD ratio in the present study at 78.2±2.6% was greater and more varied than the Bolton’s anterior ratio. This might be influenced by the fact that Bolton’s sample had normal occlusion while, only 4.5% of the present sample presented with normal occlusion. Alternatively, the overall TSD ratio in the present group found to be similar to the Bolton overall ratio [91.3%, SD=1.91], but with greater variation [SD=2.1]. This is similar to the findings reported by several researchers (5,11,25). Moreover, there was no significant difference in TSD in the anterior and the overall ratio between males and females as noted in some earlier studies (2-4,12,13,16,18,24).

The frequency of anterior and overall mean ratios with significant deviation of more than 2 SD comprised 3% and 4.2% of the total sample, respectively, which are among the lowest reported in the literature. The existence of the anterior ratio of more than 2 SD from Bolton’s mean in the present sample [3.3%] is much lower than those published figures for Yemenis (2) [14.2%], Dominican Americans (25) [28%], Americans (26) [30.6%], Peruvian (11) [20.5%], Jordanians (3) [23.7%], and Japanese (27) [21.6%]. The occurrence of clinically significant overall TSD ratio outside 2 SD from Bolton’s means in the current study was 4.2%. This value is lower than reported figures for Turkish subjects (13) [48%], Yemeni’s (2) [14.2%], Dominican Americans (25) [11%], Jordanians (3) [9.5%], Japanese (27) [8.3%], and Peruvian (11) [5%]. Thus, the present results reveal that Bolton ratios can be applied to Libyan subjects. However, the present study is considered as a preliminary study and additional nationwide research with a sufficient number of Libyan subjects in general, and each category of malocclusion, may be required to obtain a clearer picture about TSD in Libyan population across the whole country.

Unfortunately, the association between each malocclusion category and TSD could not be investigated in the present study as the number of the recruited individuals with Class II division 2 and Class III malocclusions were not enough to undertake comparative analysis. However, the present cross-sectional study was the first TSD published data for the Libyan population. Further, TSDs were common in orthodontic populations and it has been suggested that these were evenly distributed among sex, ethnicity, and malocclusion category, with some exceptions (28-30).

## Conclusions

• Males showed significantly wider MD tooth width compared to females, except for the maxillary first premolars and mandibular central incisors.

• There were significant differences between the paired tooth measurements except for the maxillary and mandibular central and lateral incisors as well as mandibular canines.

• There was no significant difference in TSD [anterior and overall ratios] between males and females.

• The frequency of anterior and overall mean ratios with significant deviation [> 2 SD] comprised 3% and 4.2% of the total sample, respectively.
